# Microbial Ecology for Engineering Biology

**DOI:** 10.1098/rsfs.2023.0027

**Published:** 2023-06-09

**Authors:** Thomas Curtis, Jane Fowler

**Affiliations:** ^1^ School of Engineering, Newcastle University, Newcastle NE1 7RU, UK; ^2^ Biology, Simon Fraser University, Burnaby, British Columbia V5A 1S6, Canada

This edition of *Interface Focus* showcases an emerging new frontier in the engineering of open microbial systems that was the subject of a Royal Society Meeting on Microbial Ecology for Engineering Biology held in spring 2022. This new frontier is being opened by the very exciting new developments in microbial ecology. Advances driven by ongoing revolutions in molecular methods, ecological theory and computing power. This expansion cannot come soon enough. Climate change and the need for a sustainable circular economy call for new and better solutions to society's needs. Those needs are to be found in both traditional applications of engineering and microbial ecology, such as clean soils and water, as well as new applications, such as greenhouse gas abatement and geotechnical engineering.

This may sound ambitious. It is. But that ambition sits firmly within the tradition of our nineteenth-century forebears who in 1825 defined engineering as ‘*the art of directing the great sources of power in Nature for the use and benefit of mankind*’. Tredgold's definition was aspirational, but his ambition was well founded. His ‘source of power in Nature’ was physics. Our nineteenth-century predecessors learnt how to synthesize Newtonian science, new materials, mathematics and their own innate ingenuity to build roads, railways, bridges, buildings and harbours that transformed countries and economies across the world.

We need a similar transformation today. Our ‘source of power in Nature’ is biology, especially microbiology. Our science is microbial ecology, and our materials are the 10^11^ taxa that have evolved over the past 4.5 billion years. The power of microbial communities does not lie in any single taxon (as so many think), rather the power arises from the numbers of, and the interactions between, the many species involved. These interactions are themselves affected by chemical, physical and biological contexts. The power in other words is an emergent property of the collective. The scale of that power is incredible and indisputable as it drives the global biogeochemical cycles that support life on Earth.

However, at present, our ability to capture that power is limited. Limited by our grasp of science, limited by the time it takes to develop new technologies, limited by the engagement with practioners, and most especially, limited by the culture of contemporary science and engineering.

The focus of the meeting was, naturally enough, on the many ongoing scientific advances: conceptualizing the design process, defining the basic ecological and physical processes, characterizing the key organisms and simulating ecosystems. Much, but not all, of the excellent work we saw is represented in this volume. Moreover, there was much creative disagreement about the best use of the tools, the most appropriate theories and the most important applications. This is as it should be.

But we are interested in engineering, not science for science's sake. In the meeting, it became increasingly clear that there are many ways to integrate the science to create solutions. And the right ones will be the ones that work. With time, not money, being our most precious resource, we should be pursuing multiple multi-disciplinary avenues in parallel, *in vivo* and *in silico*. Failure will be inevitable, but powerful, if that failure is documented, disseminated, analysed and, where possible, obviated. (Success and serendipity will hopefully also occur, but success is rarely kept a secret.)

Such an approach requires a change in our research culture. No one laboratory or research group has the skills we need to create new solutions quickly. We must work together. Although the reward structures in academia still focus on the individual, often the principal investigator (PI). We must be valued, and funded, for collegiality and teamwork. Effective teams are places where all have a voice. There may be multiple paths to effective solutions so a candid, open, tolerant (tolerant to dissent, tolerant to failure) culture must be promoted.

We are still some way from this ideal in both academia and research funding bodies. For example, in the UK at the time of writing, engineering biology is, inexplicably and narrowly, equated exclusively with synthetic biology, and a naive Panglossian expectation that the synbio paradigm will generate all the solutions we require. However, there are also grounds for optimism. Team and mission-based approaches are promoted by several national and international research bodies. A new generation of researchers is much more willing to work in teams, more universities are embracing their research culture as their superpower. Tangible success by this new generation will create a virtuous circle where the success of an improved research culture will strengthen and deepen that culture leading to more success, more investment in solutions and better ways to work together. We can, we will and we must emulate and exceed the successes of Tredgold's generation.

Success of course, has many parents (while failure is an orphan). When that success comes, the authors and editors of the papers in this journal, the attendees at the meeting and the Royal Society itself should proudly, and rightly, claim parenthood.

